# Diabetes and Foot Health Among South Asian People Seeking Asylum in the United Kingdom: A Theory‐Informed Scoping Review

**DOI:** 10.1111/hex.70728

**Published:** 2026-06-18

**Authors:** Nadera Assim, Catherine Bowen, Michelle Myall

**Affiliations:** ^1^ Faculty of Environmental and Life Sciences (FELS) School of Health Sciences Southampton UK

**Keywords:** diabetes stigma, foot health, intersectionality, patient and public involvement, people seeking asylum, postmigration, type 2 diabetes mellitus

## Abstract

**Background:**

Globally, it is estimated that, as of June 2025, 8.42 million people were seeking asylum due to persecution and political instability. Those seeking protection in other countries can present with undiagnosed complex health needs, including type 2 diabetes mellitus. Yet diabetes related foot health and footwear remain poorly understood in this population. This scoping review mapped evidence on diabetes, foot health and footwear among adults seeking asylum in high‐income countries, with attention to postmigration factors and diabetes‐related stigma.

**Methods:**

Arksey and O'Malley's scoping review framework guided the review. Levac et al. informed refinements. Reporting followed PRISMA Scoping Review standards. Searches of CINAHL, MEDLINE, PubMed and Web of Science covered January 2005 to November 2025, supplemented by grey literature. Records were screened to predefined criteria, data were charted using a structured extraction form and findings were synthesised thematically using the socioecological model and intersectionality.

**Findings:**

Eight papers were included from the United Kingdom, Australia, France and Belgium. Diabetes was noted, but asylum conditions were seldom linked to diabetes management, and stigma was rarely discussed. Foot health and footwear were largely absent. No study applied an explicit intersectional framework, despite overlapping influences of immigration status, poverty, gender and housing.

**Conclusions:**

Existing evidence remains limited, offering little insight into how postmigration conditions shape diabetes‐related foot health among people seeking asylum. Further research is needed that is structurally informed and community‐engaged. It should centre lived experience and treat stigma as relevant to diabetes care, while bringing foot health into view.

**Patient or Public Contribution:**

People with lived experience of seeking asylum contributed to this scoping review through a Community Advisory Group established as part of the wider study. Some members also had experience of living.

**Abbreviations**
CAG: Community Advisory Group
CASP: critical appraisal skills programme
GP: general practitioner
JBI: Joanna Briggs Institute
NCCDH: National Collaborating Centre for Determinants of Health
NHS: National Health Service
PCC: population, concept and context
PRISMA ScR: preferred reporting items for systematic reviews and meta analyses extension for scoping reviews
SEM: socioecological model
T2DM: type 2 diabetes mellitus
UK: United Kingdom
UNHCR: United Nations High Commissioner for Refugees
WHO: World Health Organization

## 1 Introduction

By mid‐2025, more than 117 million people worldwide had been forcibly displaced due to persecution, conflict, violence and human rights violations, including more than 8 million people seeking asylum across international borders [1]. This review focuses on South Asian populations because, as a region, it carries a substantial burden of type 2 diabetes mellitus (T2DM) [2, 3], and individuals from several South Asian countries represent a significant proportion of people seeking asylum in the United Kingdom [4]. These patterns of displacement occur within wider political, environmental and economic pressures described as a global polycrisis, in which instability, inequality and climate stress interact to concentrate harm among populations experiencing poverty, displacement and insecure legal status [5, 6, 7].

South Asia consists of countries that together account for approximately one quarter of the world's population and continue to experience political instability, environmental vulnerability and underinvestment in healthcare systems. David et al. [8, 9], public health expenditure across much of the region remains below 5% of gross domestic product, contributing to fragmented service provision and persistent challenges in managing chronic disease [10, 11]. Within this context, T2DM has increased rapidly, and a substantial proportion of cases remain undiagnosed or inadequately managed [2, 12, 13]. When individuals migrate in search of safety, these unmet health needs often accompany them into new healthcare environments where legal status, housing insecurity, financial restriction and unfamiliar health systems shape opportunities for treatment and ongoing care [14, 15, 16].

The United Kingdom is an important context in which to examine these issues. Within international law, a person seeking asylum is awaiting a decision on a protection claim, whereas a refugee has had that claim recognised under the 1951 Refugee Convention [17]. Legal status influences access to housing, welfare support and healthcare services. In the year ending December 2025, a total of 100,625 people claimed asylum in the United Kingdom [18]. The five largest nationality groups included Pakistanis, Eritreans, Iranians, Afghans and Bangladeshis, together representing 39% of all applicants [18]. South Asian countries alone accounted for 31,341 asylum claims, equivalent to approximately 31% of the total [18].

The significance of this demographic profile becomes clear when considered alongside diabetes epidemiology. Globally, more than 589 million people live with diabetes, and the prevalence continues to increase. In the United Kingdom, more than 4.6 million people have a confirmed diagnosis, the majority with T2DM [19, 20]. Research consistently shows that T2DM occurs at a higher prevalence among South Asian populations than among white British populations, reflecting complex interactions between social conditions, healthcare access, environmental exposures and long‐term disease management rather than biological difference alone [12, 13, 21, 22].

Despite this context, asylum health assessments in the United Kingdom have historically prioritised infectious disease surveillance rather than the early detection and sustained management of chronic illness [23]. As a result, individuals living with T2DM may enter healthcare systems where structural barriers limit continuity of care.

### 1.1 Migration and Health Access

Migration has increasingly been recognised as a social determinant of health because immigration status, welfare eligibility, housing conditions and healthcare entitlement shape access to care and the continuity of treatment [24, 25]. For people seeking asylum, these structural factors often create complex barriers to engaging with healthcare. Housing insecurity, restricted income, language barriers and uncertainty regarding legal status can disrupt treatment routines and limit access to preventive services [26].

Psychosocial stress, disrupted social networks and reduced community support may further complicate disease management following migration [27, 28]. Within some South Asian communities, additional challenges arise through diabetes stigma, secrecy surrounding illness and concerns about social reputation that may discourage disclosure and delay help‐seeking [29, 30, 31]. These experiences intersect with wider structural inequalities within healthcare systems, including racialised barriers, stereotyping and institutional discrimination that can influence diagnosis, treatment pathways and quality of care [32, 33, 34, 35, 36].

Although people seeking asylum in the United Kingdom are entitled to access National Health Service (NHS) primary care and, in most circumstances, secondary care, legal entitlement alone does not guarantee meaningful access [37]. Awareness of entitlements remains uneven and practical engagement with healthcare services may be shaped by bureaucratic complexity, communication barriers and limited culturally responsive provision [38, 39]. Research indicates that initial health assessments often prioritise communicable disease control and acute health needs rather than the identification and management of chronic illness [40, 41].

Collectively, these barriers may delay T2DM diagnosis among people seeking asylum and disrupt access to ongoing monitoring. Such disruption can weaken preventive care pathways and increase the risk that diabetes related complications, including foot disease, remain unidentified until they become more severe.

### 1.2 T2DM and Associated Complications

T2DM is associated with a range of serious complications affecting multiple organ systems. Among these, diabetic foot disease represents a major cause of avoidable hospital admissions and long‐term disability. Complications may include foot ulceration, infection, lower‐limb amputation, reduced mobility and premature mortality [42, 43, 44, 45].

Clinical guidance emphasises prevention through regular screening, structured education, appropriate footwear, multidisciplinary care and sustained engagement with healthcare services [46, 47]. However, these recommendations assume stable living conditions and reliable access to healthcare. For people seeking asylum, these assumptions may not hold. The management of T2DM can be disrupted by insecure accommodation, food insecurity, language barriers, financial restriction and experiences of discrimination [48, 49].

### 1.3 Evidence Gaps in Asylum Health Research

Research on asylum health has documented the effects of insecure housing, limited income, legal uncertainty and restricted healthcare access. However, much less is known about how these conditions shape the diagnosis and management of long‐term conditions such as T2DM.

An overview of systematic reviews examining the health and social care needs of adults seeking asylum in high‐income countries found that existing literature is dominated by research on mental health, access barriers, housing insecurity and communication challenges, while chronic disease management receives comparatively little attention [50]. Similarly, a scoping review of migrant health research conducted in Scotland identified very limited investigation of noncommunicable diseases such as T2DM despite the frequent inclusion of asylum‐seeking populations within broader migrant health research [51]. The absence of research examining diabetes‐related foot health within asylum populations is therefore notable.

This gap exists despite the recognised burden of T2DM among South Asian communities and the substantial representation of South Asian countries among people seeking asylum in the United Kingdom. Within wider UK health scholarship, foot health itself has been described as a persistently marginalised dimension of care shaped by intersecting social determinants including ethnicity, socioeconomic position, chronic illness and access to appropriate footwear [52].

Healthcare access and chronic disease management are shaped by the organisation and funding of national health systems. High‐income countries typically allocate greater public expenditure to healthcare and maintain more extensive service infrastructure than middle‐income settings, influencing how long‐term conditions such as T2DM are diagnosed and managed [53]. The United Kingdom shares these structural characteristics, making it a relevant context for examining how asylum systems and healthcare organisations shape access to care within comparable high‐income settings.

This novel scoping review forms part of a wider study examining the experiences of South Asian people living with T2DM seeking asylum in the United Kingdom. The review aims to map the existing research evidence focusing on T2DM and related foot health among people seeking asylum and to identify gaps, silences and assumptions within the current literature.

## 2 Review Design and Methods

### 2.1 Theoretical Frameworks

This scoping review is theoretically anchored in the socioecological model (SEM) and intersectionality, enabling a novel mapping of T2DM‐related foot health among people seeking asylum across interlocking levels of influence and power [54, 55]. The SEM was used to situate individual experiences within interpersonal relations, service organisation, community environments and wider policy contexts, rather than locating T2DM management in personal behaviour alone [56, 57]. This approach responds to critiques that behaviour‐centred models can obscure structural drivers of inequality and reproduce blame and stigma when people anticipate judgement within services [58, 59], while retaining the model's established relevance for multilevel health promotion in contexts of instability and limited options [60, 61]. Intersectionality extended this framework by examining how race, gender, class, disability and immigration status interact through institutions and policy to shape exclusion, language access and bureaucratic control within T2DM and foot care [55, 62, 63].

### 2.2 Methods

Migrant health evidence is hard to synthesise because definitions and methods vary and undocumented populations are often missing from datasets, making a systematic review unsuitable [64, 65]. As an underdeveloped field, a scoping review was undertaken to map existing evidence on foot health among South Asian people seeking asylum with T2DM.

**Figure 1:**
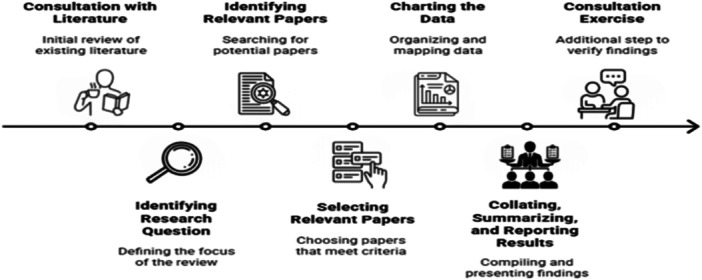
Iterative scoping review process adapted from Arksey and O'Malley [66] and Levac et al. [68].

The review followed Arksey and O'Malley's framework, incorporating refinements by Levac et al. and guidance from the Joanna Briggs Institute (JBI), and was reported in accordance with preferred reporting items for systematic reviews and meta analyses extension for scoping reviews (PRISMA‐ScR) standards [66, 67, 68, 69]. (Figure 1) The completed PRISMA‐ScR checklist is provided in Supporting Information S1: Appendix S1.

A preliminary search of the Cochrane Library, CINAHL, the JBI Database, the Open Science Framework, PROSPERO and Medline did not identify any reviews on this topic.

#### 2.2.1 Stage 1: Consulting the Literature

Consultation with literature and knowledge holders, often treated as an additional stage in scoping review methodology, was positioned as central to this review [66, 68, 70]. Initial engagement with the literature tested feasibility and clarified conceptual boundaries around T2DM, foot health and postmigration experiences among people seeking asylum with T2DM, revealing limited evidence on South Asian populations, minimal attention to foot health or footwear and little engagement with diabetes stigma. The scope was therefore broadened to adults seeking asylum with T2DM in high‐income countries, while retaining a focus on postmigration conditions.

This decision reflected the limited empirical literature on T2DM among people seeking asylum. Studies conducted in high‐income host countries, including the United Kingdom, Australia, France and Belgium, were included because these settings share some broadly similar structural features in asylum governance and healthcare systems that shape access to care [24, 25]. While service provision varies, these countries operate within policy environments where legal status and entitlement influence continuity of care, making them relevant for comparison with the UK context [71].

#### 2.2.2 Stage 2: Identifying the Research Question

To guide the review and align with its exploratory aim, the following primary research question was formulated: *What is known about how postmigration factors shape the health experiences and diabetes‐related self‐management challenges of people seeking asylum in high‐income countries?*

The research subquestions were:

1. What structural, cultural and systemic factors influence T2DM care among people seeking asylum in high‐income countries?
2. To what extent, if any, is diabetes stigma acknowledged or examined?
3. How are foot health and footwear practices reported in the literature?

#### 2.2.3 Stage 3: Identifying the Relevant Studies

The search strategy followed JBI guidance and used the Population, Concept and Context (PCC) framework to structure terms [65, 69]. A preliminary search of the Cochrane Library, CINAHL, the JBI Database, the Open Science Framework, PROSPERO and Medline identified no existing reviews. Full searches were undertaken in CINAHL, MEDLINE, PubMed and Web of Science from January 2005 to November 2025. The review was conceptually informed by a social determinant of health perspective, which emphasises the role of social and structural conditions in shaping the risk and management of long‐term conditions [72]. Search terms combined population labels with concepts relating to diabetes, foot health and stigma, alongside contextual terms capturing postmigration conditions and health inequalities (Supporting Information S2: Appendix S2). Two specialist librarians supported refinement using Boolean operators, truncation and controlled vocabulary.

Database searches were supplemented through reference list screening, citation tracking in Google Scholar and targeted searches of organisational websites, including the British Red Cross, Doctors of the World UK, the Refugee Council and the World Health Organization. Inclusion and exclusion criteria were developed iteratively. Eligibility criteria are outlined in Table 1. Inclusion and exclusion criteria were defined according to the review question and the population and setting under investigation. According to guidance by Dekkers et al. [73], eligibility criteria should follow logically from the review question and specify the relevant population or subjects included in the analysis. Only English‐language papers were screened, balancing feasibility with concerns about the accuracy of automated translation tools [74, 75]. All records were collated, de‐duplicated and screened by title and abstract, followed by full‐text review where eligibility was unclear, with decisions made collaboratively within the research team.

**Table 1:** Inclusion and exclusion criteria.

Inclusion	Exclusion
Papers published from 2005 to present Human participants Adults over 18 years old Papers with South Asian people seeking asylum with T2DM Papers with people seeking asylum with T2DM Papers that report one or more postmigration factor Papers that investigated the relationship between postmigration factors and diabetic foot disease profiles Papers that investigated diabetes stigma in people seeking asylum Papers that reported footwear in people seeking asylum Peer‐reviewed papers and grey literature	Papers published in languages other than English were excluded, as Google Translate was deemed unreliable and potentially inaccurate for research purposes Papers on internally displaced persons and undocumented migrants Papers that reported interventions Papers that assessed risk factors Papers reporting studies conducted exclusively in detention centres or refugee camps were excluded because these represent highly controlled institutional environments that differ from community‐based asylum accommodation systems in high‐income countries. Papers investigating gestational diabetes, Type 1 diabetes and prediabetes Papers that reported solely on comorbidities or health problems Papers that grouped refugees, migrants and people seeking asylum Full text not accessible Systematic Reviews

#### 2.2.4 Stage 4: Study Selection

**Figure 2:**
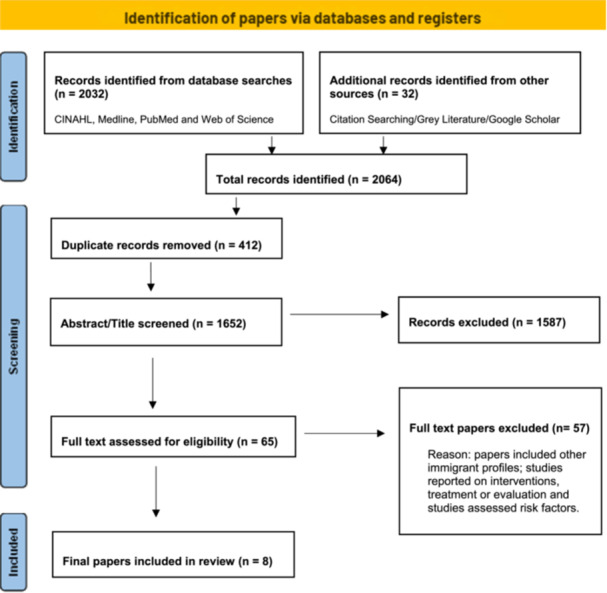
PRISMA‐ScR flow diagram, adapted from Tricco et al. [67].

Titles, then abstracts of all records identified through database and hand searches, were screened against the predefined inclusion and exclusion criteria. Full texts were obtained whenever eligibility could not be determined from the abstract. An inductive approach was then used to extract data from the final set of included papers. Each paper was read in full and charted in relation to the core focus of the review, with attention to both reported content and notable absences. Findings are reported in line with the PRISMA‐ScR [67] (Figure 2).

#### 2.2.5 Stage 5: Charting the Data

Data were extracted into a structured charting table (Supporting Information S3: Appendix S3) [76, 77, 78, 79, 80, 81, 82, 83]. Charting was conducted manually and cross‐checked. Included papers varied markedly in design, depth, population focus and analytic emphasis; some examined T2DM management directly in asylum contexts, whereas others mentioned T2DM only briefly or within broader accounts of long‐term illness. None applied a formal intersectional framework, although several acknowledged overlapping influences such as gender, immigration status, poverty and access barriers.

Formal quality appraisal was not used for exclusion, consistent with scoping review guidance and the aim of mapping rather than grading evidence [69]. Given the small, fragmented literature on people seeking asylum with T2DM, strengths and limitations were recorded to support cautious, critical interpretation.

Formal critical appraisal tools, such as CASP, were not used, consistent with scoping review guidance [84]. However, study design, scope and reported limitations were considered during synthesis and interpretation of the findings.

#### 2.2.6 Stage 6: Collating, Interpreting and Reporting Findings

Findings reported in each included paper were read in full. Text describing T2DM experiences and postmigration conditions was coded to identify recurring patterns across the studies. Initial codes were generated from reported findings within each paper.

Codes were then compared across studies and grouped into broader thematic categories through iterative review within the research team. This process enabled patterns to be identified across sources that varied in design, population focus and analytic depth.

The thematic categories were subsequently organised according to the levels of the SEM to identify the level at which each influence operated. Codes describing knowledge, behaviour or personal experience were categorised at the individual level. Codes describing immediate living environments or interpersonal relationships were mapped to the microsystem. Codes relating to interactions between services were categorised within the mesosystem. Community resources and environmental conditions were allocated to the exosystem, while immigration policy, welfare structures and legal frameworks were mapped to the macrosystem. Time‐related processes affecting continuity of care were categorised within the chronosystem.

An example illustrating how extracted findings were coded and mapped to SEM levels is provided in Supporting Information S4: Appendix S4.

#### 2.2.7 Stage 7: Consulting Knowledge Holders

Although consultation is often an optional component of scoping reviews [66, 68], it was considered an essential stage of this review. After the initial thematic analysis, a plain‐language summary of findings was shared with the Community Advisory Group (CAG) to explore whether themes resonated with lived and professional experience and to prompt collective reflection.

#### 2.2.8 Patient and Public Involvement and Engagement

Patient and public involvement and engagement were incorporated through a CAG established as part of the wider study. The group was formed through collaboration with community organisations and local networks supporting people seeking asylum, identified through existing partnerships and community engagement activities. Organisations were approached directly by the research team and invited to share information about the study with individuals who may wish to take part, with members recruited on a voluntary basis. The group included people with lived experience of seeking asylum, and members with experience of living with T2DM or caring for someone with T2DM. They contributed as public partners, informing interpretation. CAG input occurred after completion of the evidence synthesis and was used to sense‐check interpretations of gaps and silences, rather than to shape the review question or search strategy. Following the preliminary synthesis, a plain‐language summary of the findings was shared with the group. Reflections were recorded in field notes and used to refine how the synthesis represented the evidence in relation to lived experience.

## 3 Findings

### 3.1 Paper Characteristics

**Figure 3:**
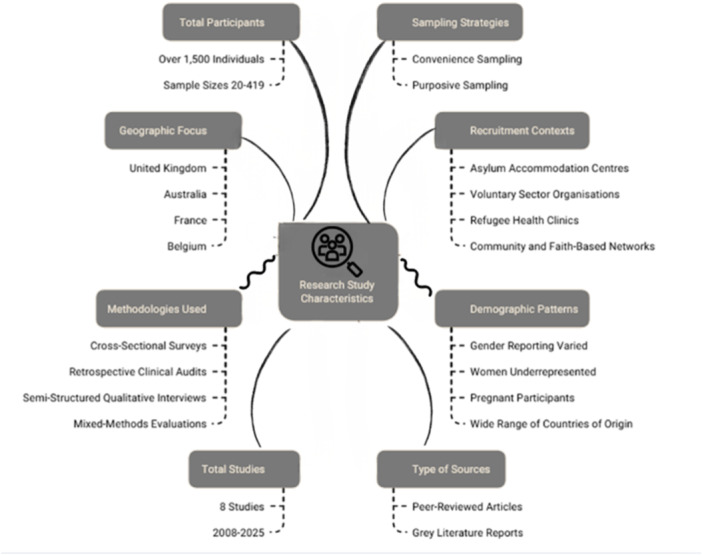
Characteristics of included papers.

This scoping review included eight sources published between 2008 and 2025, with most published after 2020. Five sources were peer‐reviewed articles [77, 79, 80, 82, 83]; and three were grey literature reports from the United Kingdom [76, 78, 81] (Figure 3). Overall, the included studies were conducted in the United Kingdom, Australia, France and Belgium. Sample sizes ranged from 20 to 419, with recruitment primarily through asylum accommodation, voluntary and community organisations, and refugee health services. Most studies focused primarily on men. Women, including pregnant women, were frequently underrepresented. Participants’ origins were diverse, spanning South Asia, the Middle East and several African regions, although reporting varied between detailed country lists and broader regional groupings. None of the included studies centred on South Asian people seeking asylum as a distinct population group, although some included participants from South Asian countries within broader asylum‐seeking samples.

A range of methods were utilised, including cross‐sectional surveys, retrospective clinical audits, semi‐structured qualitative interviews and mixed‐methods evaluations, typically using convenience or purposive sampling. While this reflects methodological breadth, the evidence base remains primarily descriptive and context‐specific.

Participants with T2DM were reported in six papers, with only limited reference to foot‐related issues, and none examined footwear practices or their social meanings. Stigma and overlapping influences such as gender, immigration status and housing conditions were occasionally noted, but none of the studies applied an explicit intersectional framework. Evidence relating to T2DM appears only marginally within the broader health literature on people seeking asylum [79, 80, 82, 83], with only limited work directly examining T2DM management [77]. No studies addressed foot health, foot complications, footwear or diabetes‐related foot self‐management.

### 3.2 Mapping Findings Across the SEM

**Figure 4:**
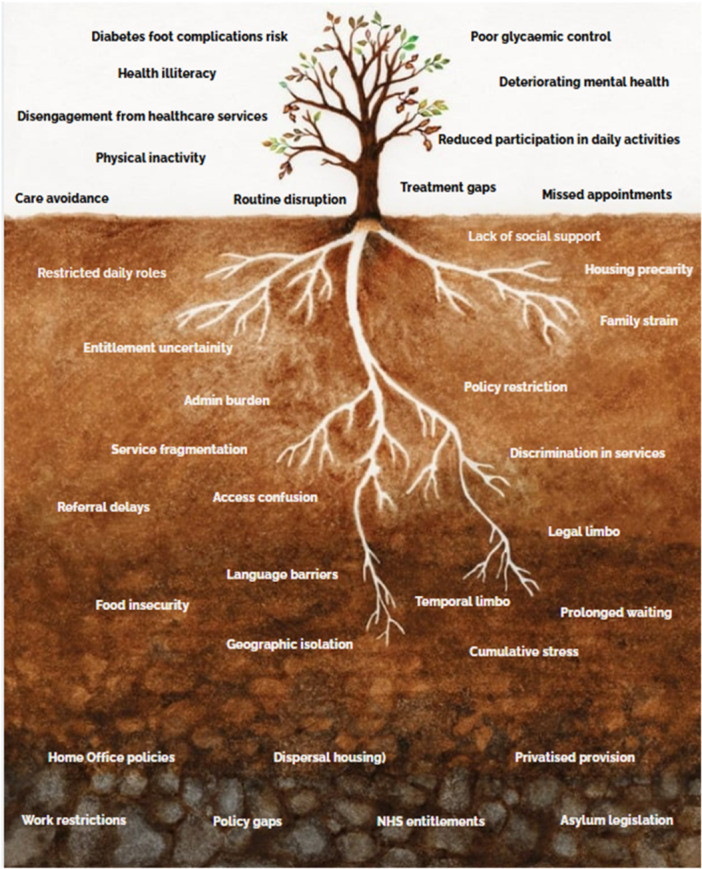
Adapted tree model (and legend) mapping health outcomes, behaviours, conditions and structural drivers shaping T2DM care in asylum contexts [85]. Legend: Leaves represent individual‐level outcomes; the trunk reflects individual experiences within immediate living environments; roots capture microsystem, mesosystem and exosystem influences; and soil denotes macrosystem and chronosystem conditions.
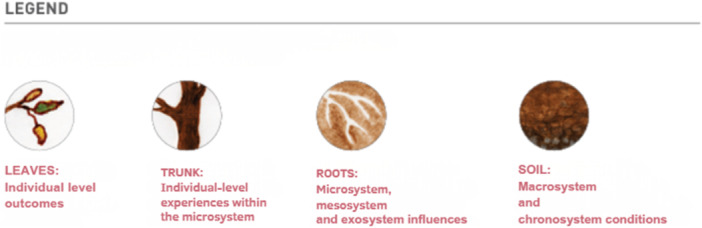

Findings were organised using an adapted [85] SEM to show how postmigration conditions shape foot health inequalities across six levels: individual, interpersonal, organisational (meso), community (exo), structural or macro and temporal (chronosystem) (Figure 4). The individual level was used to situate lived experience within wider systems, rather than to assign responsibility to people seeking asylum. This framework supported analysis of both immediate factors, such as health literacy and service access, and the institutional, policy and time‐related conditions that structure care, with the synthesis presented thematically from the individual level through to the chronosystem to illustrate how waiting and instability accumulate across the asylum journey.

### 3.3 Individual Level

Across included papers, individual‐level accounts emphasised psychological distress and disrupted self‐management. Participants reported posttraumatic stress, anxiety and depression linked to premigration trauma, forced displacement and occupational deprivation [78, 82, 83]. Limited health literacy and uncertainty about T2DM or prediabetes management, including the role of physical activity, were also described [80]. Chronic stress, poor sleep and loss of routine further undermined day‐to‐day T2DM management [77]. Correa‐Velez et al. [79] similarly reported low mood, social isolation and the strain of navigating unfamiliar systems, despite not focusing on T2DM.

### 3.4 Microsystem

Accommodation and other immediate environments were often depicted as unsafe, isolating and neglectful [76, 78, 81, 83]. Across studies, participants described overcrowding, limited privacy and strained relationships with staff or other residents [78, 81, 83]. The Refugee Council [76] reported that hotel confinement could involve restricted movement, inadequate nutrition and a sense of surveillance. Women with children described difficulty meeting their own health needs within confined living arrangements [80]. Language barriers, mistrust and high levels of mental distress further shaped interpersonal dynamics, alongside occasional safeguarding failures that increased risk and eroded confidence in services [77].

### 3.5 Mesosystem

At the mesosystem level, weak coordination between services disrupted care. Participants reported barriers to General Practitioner (GP) registration, access to prescriptions and continuity of care after relocation [79, 81]. Poor communication between housing providers, health services and voluntary sector organisations contributed to medication gaps, missed referrals and delayed follow‐up [77]. This fragmentation increased reliance on volunteers and charities, while accommodation staff were sometimes unclear about entitlements, leaving urgent needs unmet [76, 83].

### 3.6 Exosystem

At the exosystem level, community infrastructure and local environments shaped everyday health. Participants were often placed in isolated areas with limited transport, green space or culturally familiar services [79, 80, 81]. Institutional accommodation frequently lacked basic amenities, including cooking facilities, and food provision was described as limited, nutritionally poor, culturally inappropriate and linked to difficulties maintaining glycaemic control [76, 77]. Digital exclusion, limited translation support and weak orientation to local services compounded these barriers [77, 81].

### 3.7 Macrosystem

At the structural level, national policy shaped whether care was accessed or avoided. In the United Kingdom, dispersal and the inability to choose where to live produced instability that undermined health‐seeking [78, 81, 83].

The Refugee Council [76] further reported confusion about entitlements, fear of charging and racism within NHS settings as deterrents to attendance. In Belgium, unclear eligibility rules and administrative barriers disrupted T2DM care pathways [77], while in Australia, welfare exclusions contributed to income insecurity, poorer diet and interrupted medication access [79].

### 3.8 Chronosystem

The chronosystem highlighted how time and uncertainty accumulated to shape health. Prolonged waiting, repeated relocations and unresolved legal status contributed to disengagement from care and worsening mental health [77, 78, 83]. Jones et al. [81] reported that people who had initially been proactive became increasingly withdrawn as disruptions persisted and planning became impossible. This sense of suspension, with restricted access to education, work and consistent healthcare, was also documented by the Refugee Council [76]. Time in this context functioned not as a neutral backdrop but as an active driver of harm.

## 4 Discussion

This scoping review examined how postmigration conditions shape T2DM management among people seeking asylum in high‐income countries, with particular attention to T2DM and foot health. Using a socioecological lens, the review traced how individual experiences of illness are produced within wider systems of housing, welfare, healthcare and immigration control. Alongside synthesis of the included literature, insights from the CAG informed interpretation by interrogating how published accounts align with lived experience and where important dimensions remain underexplored. As one CAG member reflected, ‘They research migrants or refugees, but they do not ask people like us. We are just assumed’. This observation captures a central tension running through the evidence base and frames the discussion that follows.

### 4.1 Reframing Individual Responsibility

None of the included papers examined diabetes‐related foot self‐care directly. Instead, they described stress, uncertainty and disruption as undermining engagement with T2DM management more broadly [77, 80, 82, 83]. Psychological and physical distress linked to poverty, housing instability and loss of autonomy was repeatedly identified, echoing wider work on structural vulnerability in migrant health [86]. Within this context, disengagement from care appears less as a matter of motivation and more as a response to environments that make sustained self‐management difficult.

Food insecurity featured prominently across accounts. Although diet was not measured quantitatively in the included studies, wider evidence describes restricted food choice, limited culturally appropriate provision and poor access to cooking facilities within institutional accommodation [87, 88]. These conditions are clinically relevant given the association between food insecurity and poorer glycaemic control among adults with T2DM [89]. Evidence from the wider diabetic foot literature further demonstrates that social deprivation and limited access to care are closely linked to the development and progression of foot disease, reinforcing the interpretation that foot health inequalities are structurally produced rather than behaviourally driven [90]. This aligns with broader T2DM scholarship showing that housing insecurity, food access and healthcare navigation fundamentally shape outcomes, limiting the effectiveness of behaviour‐focused self‐management approaches when structural support is absent [91].

Health literacy was discussed in several papers but was closely linked to postmigration conditions. Participants described uncertainty about managing T2DM while navigating unfamiliar systems, language barriers and housing instability [80, 82]. Although beliefs about the inevitability of T2DM have been reported in some South Asian communities, these interpretations sit alongside repeated experiences of exclusion from care, suggesting withdrawal rather than fatalism [31, 92, 93].

At the individual level, women's caregiving responsibilities reduced their practical capacity for self‐care and care‐seeking. Women were positioned as caregivers with limited autonomy over health decisions, transport and attendance at appointments [80, 94]. These accounts emphasise that T2DM management is relational and culturally embedded, and that behavioural framings of ‘non‐compliance’ obscure how responsibility is negotiated within households and structured by wider systems. Overall, the evidence indicates that education alone is insufficient without material security, privacy, interpretation and culturally meaningful support [38].

### 4.2 Disconnection and Isolation in Relationships and Services

At the interpersonal and institutional levels, fragmented care and poor communication were consistently evident. Interpreter provision was one of the most persistent barriers. Participants frequently relied on informal interpreting and translation because formal services were unavailable or unreliable, undermining understanding, confidentiality and trust [77, 80, 81]. This aligns with evidence that language access is integral to equitable care rather than a discretionary support [95, 96].

Institutional fragmentation further disrupted T2DM care. Difficulties registering with primary care, interruptions to medication and abrupt relocations were commonly reported [77, 78, 81]. These disruptions were emotionally taxing and reinforced perceptions of invisibility, particularly where individuals were required to repeatedly recount their histories across disconnected services [97]. The concept of ‘missingness’, described by the South Asian Health Foundation [98], captures how minoritised patients may remain unseen despite formal entitlement. Within this context, reduced engagement with care appears to be a rational response to systems perceived as unreliable and unsafe.

### 4.3 Structural Barriers to Access

At the structural level, accommodation, dispersal policies and immigration governance emerged as decisive influences on T2DM and foot health. Housing functioned as an active driver of health risk rather than a passive backdrop. Overcrowding, poor‐quality accommodation and lack of accessibility undermined hygiene, medication adherence and attendance at appointments [78, 80, 81, 83]. This aligns with wider public health evidence that housing security is a core condition for health equity [99, 100].

Dispersal policies disrupted continuity of care through repeated relocations, fracturing GP registration and specialist follow‐up [76]. Comparable patterns have been reported internationally, including in Belgium and Greece, where legal entitlements and housing stability were directly associated with T2DM outcomes [77, 101].

While the eight included studies did not directly examine structural racism, the patterns they reported can be understood in light of wider evidence demonstrating how immigration policy, housing allocation and service design contribute to racialised health inequities. Concepts such as structural racism, allostatic load and weathering were therefore drawn from the broader literature to contextualise and interpret the findings, rather than being explicitly evidenced within the included studies themselves.

Evidence links immigration control, housing allocation, discrimination and service design to the production of health inequities among people seeking asylum [102, 103]. Agarwal et al. [104] argue that T2DM inequities reflect predictable outcomes of structural and geographic disadvantage rather than individual behaviour, highlighting the risks of self‐management expectations divorced from upstream conditions.

### 4.4 Time, Waiting and Accumulating Harm

Time emerged as a critical postmigration influence on T2DM care. Prolonged waiting, legal uncertainty and repeated disruption compounded stress and undermined continuity [77, 78, 81, 83]. Waiting functioned as an active process through which harm accumulated. Although the included studies did not examine biological stress pathways directly, wider evidence linking prolonged asylum processes to deteriorating metabolic markers and physical health provides context for interpreting these patterns [105, 106]. This interpretation aligns with concepts of allostatic load and weathering, which describe how prolonged exposure to structural stressors can accumulate physiological harm over time [107, 108, 109].

Within this context, foot health can be understood as a consequence of disrupted and inconsistent care across the asylum process. Ongoing cycles of waiting, interruption and uncertainty shape forms of T2DM management which remain possible over time.

### 4.5 Diabetes Stigma, Footwear and Intersectionality

Stigma was rarely addressed directly in the included papers, yet surfaced indirectly through accounts of neglect, unequal treatment and erasure. Participants described medication delays, inappropriate food provision and deterioration in glycaemic control [77, 81, 83]. CAG members described feeling ‘punished just for being in the system’ and perceived primarily through immigration status rather than health need. Evidence from emergency shelter settings shows how hypoglycaemia may be misinterpreted as substance use, illustrating how institutional logics stigmatise T2DM [110]. Speight et al. [111] note the near absence of people seeking asylum from global diabetes stigma research despite clear links between stigma, distress and avoidance of care.

Exploration of footwear was not included in the papers, despite evidence linking inappropriate footwear and diabetic foot complications [47, 112]. This omission reflects a broader gap identified in humanitarian health research, where foot care for displaced populations with T2DM remains poorly documented despite clear clinical importance [113]. Evidence from lower‐ and middle‐income settings shows high prevalence of open or ill‐fitting footwear associated with poverty and limited choice [114, 115]. Reports from United Kingdom asylum accommodation, where individuals may be issued unsuitable footwear, suggest that these risks are likely to be acute, yet remain undocumented [116].

Intersectionality was not explicitly adopted in any of the eight papers, though their framings and silences underline its relevance. Barriers such as language, food insecurity, trauma and mistrust were described but rarely analysed together [77, 78, 81, 83]. Justice‐oriented scholarship has drawn attention to how foot health and chronic pain are rarely examined through anti racist lenses, even though they are closely tied to material conditions, including income, housing, exposure to racism and discrimination and the unequal organisation of care and support [52, 117]. Evidence from Canada highlights that South Asian health outcomes vary when analysed through gender and immigration status, reinforcing the need to treat people seeking asylum as heterogeneous rather than as a single category [118]. The limited visibility of women within the literature is therefore analytically significant. Wider work shows that refugee and migrant women face layered disadvantage in healthcare access and safety, shaped by gendered expectations and unequal power in clinical encounters [119, 120]. CAG testimonies underscore this point by illustrating how power can operate in the consultation through routine interaction, not only through overt exclusion. One account described attending an appointment for her own health while her husband was present, yet the clinician repeatedly directed questions and explanations to him, even though she had a higher level of English literacy. For CAG members, this prompted reflection on how assumptions about who should be addressed and believed can shape which voices are prioritised, even when language competence and formal entitlement are not in question.

### 4.6 Implications for Research, Policy and Practice

This scoping review illustrates how integrating patient or public contribution at the interpretive stage can strengthen understanding of evidence gaps in under‐researched areas of health and social care. It shows that postmigration conditions play a central role in shaping T2DM and foot health among people seeking asylum. Housing, dispersal, language access, racism, time within asylum systems, stigma and neglect of footwear intersect to determine what care becomes possible and whose needs are recognised. The evidence also highlights an analytic limitation: people seeking asylum are often treated as a homogeneous group, obscuring how risk and access vary across gender, ethnicity, disability, religion and legal status. An intersectional approach is therefore necessary to explain how policy and institutional practices generate unequal outcomes rather than simply documenting disparities [121]. For migration and health scholarship, the findings point towards research designs that attend to structural organisation rather than behavioural explanation alone. For policy and practice, they highlight the need for approaches that recognise T2DM and foot health as core components of equitable care for people seeking asylum, rather than optional add‐ons.

### 4.7 Strengths and Limitations

This scoping review is the first to map evidence on foot health among people seeking asylum with T2DM. It followed established scoping review guidance and was reported in line with PRISMA Scoping Review standards [66, 67, 68]. Consultation with the CAG after synthesis supported interpretation by grounding the conclusions in lived and professional insight.

This review was limited by a small evidence base, with only eight eligible sources. No included study examined South Asian people seeking asylum with T2DM as a distinct population group. Foot health was also seldom treated as a primary outcome.

English‐language restrictions and an inevitably incomplete grey literature search may have missed relevant evidence, particularly from refugee‐led and nongovernmental organisations. Given the focus on South Asian populations, conclusions should be treated as applicable to comparable settings rather than representative of all displaced groups.

Despite the clinical importance of diabetic foot complications, the literature included in this review offers almost no direct evidence on foot health among people seeking asylum. None of the studies examined footwear practices or foot self‐care, leaving a substantial gap in understanding both the clinical risks and the broader nonclinical implication, such as mobility, dignity, comfort and the ability to participate in daily life, that foot health holds for this population.

## 5 Conclusions

This review highlights that the conditions encountered after migration shape how T2DM is managed among people seeking asylum in high‐income countries, yet these conditions are seldom examined in depth. Foot health and footwear are sidelined mainly; stigma is only briefly addressed, and asylum research that differentiates experiences by ethnicity remains scarce. The review therefore calls for research that is structurally grounded and developed in genuine partnership with displaced communities, and that treats people seeking asylum as a heterogeneous population whose experiences of T2DM and foot health are shaped by intersecting identities and social positions, including gender, ethnicity, religion, age, legal status and disability.

## Author Contributions

**Nadera Assim:** conceptualisation, methodology, investigation, data curation, formal analysis, writing – original draft, writing – review and editing. **Catherine Bowen:** conceptualisation, methodology, formal analysis, supervision, writing – review and editing. **Michelle Myall:** conceptualisation, methodology, supervision, validation, formal analysis.

## Ethics Statement

Ethics approval was not required for this scoping review, as it involved only the analysis of published literature.

## Consent

Patient consent was not required as this study did not involve primary data collection with human participants.

## Conflicts of Interest

The authors declare no conflicts of interest.

## Supporting information

Supporting File 1

Supporting File 2

Supporting File 3

Supporting File 4

## Data Availability

Data sharing not applicable to this article as no datasets were generated or analysed during the current study.
