# Supplementary material for: Diabetes and Foot Health Among South Asian People Seeking Asylum in the United Kingdom: A Theory‐Informed Scoping Review
Source: Health Expect. 2026 Jun 18;29(3):e70728. doi: 10.1111/hex.70728 (PMC13280213; doi:10.1111/hex.70728)
Supplement: Supplementary file 2 — Supporting File 2 [file HEX-29-e70728-s001.docx]

Appendix 2

This table summarises the key concepts, related synonyms, and truncations used to construct the database search strategy.

|  |  | Search Term synonyms |  |  |  |
| --- | --- | --- | --- | --- | --- |
| Population | **Adult** | **Men, women** |  |  |  |
|  | **asylum seekers** | **Refugee, migrant, displaced person, immigrant** | **Asylum seeker* , Displace*** | **Adult asylum seeker*, Migrant*, Refugee*, Immigrant*, Displaced person, Diaspora** | **asylum seek*, refugee*, immigrant*, migrant*, displaced person*, displaced people*, uprooted person, stateless person OR “forcibly displaced’’** |
|  | **South Asia** |  | **Asia*** |  |  |
|  | **Living in the UK** | **UK residence,** |  |  |  |
|  | **Type 2 diabetes** | **Diabetes, chronic disease, non-communicable disease** | **Diabet*** | **Diabetes** |  |
| Intervention / Variable of interest / Concept | **Post migration** | **After migration, legal status, support networks, employment, work permits, immigration detention, human mobility, cultural transition,** |  |  | **post- migration, post migration* OR post – settlement** |
|  | **Diabetes related stigma** | **Diabetes stigma** | **Discrimination** |  |  |
|  | **socioeconomic factors** | **living conditions, separation from family members, support networks, policy, economics of migration, human rights, exploitation,** | **Econom*, Socioeconomic** | **Socioeconomic, Social, Economic, Education, Employment, Community Safety/Unsafe neighbourhood, Financial Insecurity, Food insecurity, Housing/homelessness/safe accommodation/affordable housing, Medical care/health/care** | **socioeconomic factors OR socioeconomic stressors** |
|  | **Foot condition** | **Feet/ Foot health, foot pain, footwear, walking, foot ulcer, persistent pain, torture** | **Foot, Feet, Wound*, Disease, Infect*, Amputation** | **Foot, Infection, Amputate** |  |
| Outcome/ Context | **Health and well-being** | **Post migration stress, refugee health, mental health, Global health, Public health, Occupational health, disease risk, diabetes risk, refugee health, self-management, foot health** | **Health, Pain, Disabled, Disability, Mobility, Footwear, Cloth*, Liv*, Fit, Quality of life, Lived experience ,Poor*, Poverty, ,Quality of life, Pain, Function** | **Quality of life, Lived experience, health, well being, Poverty, Mobility** | **health*, well -being, wellbeing, illness*, quality of life OR health related quality of life** |
