# Supplementary material for: Diabetes and Foot Health Among South Asian People Seeking Asylum in the United Kingdom: A Theory‐Informed Scoping Review
Source: Health Expect. 2026 Jun 18;29(3):e70728. doi: 10.1111/hex.70728 (PMC13280213; doi:10.1111/hex.70728)
Supplement: Supplementary file 3 — Supporting File 3 [file HEX-29-e70728-s002.docx]

Appendix 3

This table presents the key characteristics of the eight included papers, including study aims, settings, participant characteristics, methods, and reported post-migration factors.

**Characteristics of included papers**

| Authors (Year) | Country | Participants | Aim/Purpose | Methodology | Theoretical Framework | Sampling | Post Migration Factors | Diabetes/  Foot problems/  Footwear /  Diabetes stigma | Was Intersectionality considered? | Limitations |
| --- | --- | --- | --- | --- | --- | --- | --- | --- | --- | --- |
| Haith-Cooper et al., (2018) | United Kingdom (Northern England) | 36 people seeking asylum  Female - 20  Male - 16  Countries –  Bolivia, Eritrea, Ethiopia, Guinea, India, Iran, Iraq, Kashmir, Libya, Malawi, Nigeria, Pakistan, South Africa, Sri Lanka, Sudan, Syria, Zimbabwe  Age Range = 18 to 59 years old | To explore barriers and facilitators influencing uptake and adherence to physical activity among people seeking asylum in the UK.  To develop intervention strategies addressing these factors in preparation for piloting | Semi-structured group interviews | The Theoretical Domains Framework (TDF) | -Purposive sampling  -Participants were recruited via voluntary sector refugee networks and local asylum seeker support groups in four towns/cities in Northern England  -Group leaders assessed participants’ English language skills and facilitated peer interpretation to aid communication during interviews | -Stress, poverty, and the uncertainty of living conditions as an asylum seeker significantly hindered physical activity engagement.  -Lack of knowledge about local facilities and opportunities.  -Cultural norms, social isolation, childcare responsibilities, and gender-specific barriers.  -The temporary and unstable living situation reduced motivation and capability to participate in physical activity. | "I’m pre-diabetic, so, I know physical exercise is supposed to help, so, I don’t want to become diabetic next year”  "Physical health conditions were felt to influence capability to undertake physical activity and/or exercise. This included back pain, heart conditions, breathing problems, diabetes and also sports injuries." | Not explicitly used; multiple overlapping factors (gender, culture, poverty, asylum status) acknowledged via Theoretical Domains Framework | -Small sample  -Limited English skills may have restricted depth of discussion  -Findings may not represent people seeking asylum not engaged with community groups but could be transferable to similar populations |
| Khouani et al., 2022 | France | 419 people seeking asylum  Female -133  Male- 285  Unknown - 1  Geographical origin: West Africa 40%, Maghreb 16%, Central Asia 15%, Europe 12%, Middle East 12%, Rest of Africa 5%  Included less than 21 days after arrival in France | To describe the health status of people seeking asylum recently arrived in France  To investigate explanatory factors of health status among people seeking asylum | Cross-sectional observational epidemiological study  Screening of mental and somatic health via questionnaires, biological sampling, Cumulative Illness Rating Scale (CIRS), and chest X-rays  Multivariate logistic regression used to assess explanatory factors for health status | none | Participants recruited at the single reception platform for people seeking asylum | -Mental health disorders prevalent among people seeking asylum (e.g., PTSD, depression, anxiety) related to personal experiences and stress,  -Somatic diseases and personal health status (e.g., diabetes, hepatitis B),  -Sex differences influencing health status (women more likely to have somatic disease) | Diabetes was assessed via fasting blood glucose >2 g/l.  Prevalence of diabetes was reported at 1% among people seeking asylum | No | -No established psychiatric diagnosis, only screening for symptomatology  -Unable to compare health status with refugees who have been in France longer, limiting detailed understanding of post-migration effects  -Data from a single centre may limit generalizability  - Limited sample size relative to all people seeking asylum arriving during study period |
| Van Hemelrijck et al., 2022 | Belgium | 20 participants with diagnosed diabetes mellitus, all asylum-seekers living in Belgian reception centres  Female - 10  Male -10  Countries of origin include: Afghanistan (15%), Armenia (10%), Burundi (10%), Ethiopia (5%), Iraq (10%), Kosovo (5%), Lebanon (5%), Morocco (5%), Palestine (5%), Rwanda (5%), Somalia (5%), Syria (15%), Zaïre (Congo) (5%) [T4, p.5]. | To examine how displaced people with diabetes experienced managing their illness | Mixed-methods approach: Development of a bespoke interview instrument comprising closed- and open-ended questions focused on socio-demographic characteristics, migration history, diabetes diagnosis and management Interviews conducted in summer 2018 in three Belgian reception centres, facilitated by interpreters, approx. 50 minutes each Directed content analysis with coding in Nvivo 11 | Directed content analysis approach based on existing knowledge about diabetes management and migration with an emergent coding structure developed collaboratively by authors. | Purposive sampling | -Challenges maintaining diet; stress and mental health affecting self-care  -Reception centre living conditions; interactions with healthcare providers  -Connections between asylum process and healthcare access; coordination between centres and hospitals  -Reception centre policies on meals and care; asylum system healthcare provisions  -National asylum and healthcare policies; EU immigration regulations affecting access  - Cumulative stress and transitions impacting care continuity | Diabetes was discussed | No | - Small sample size  -Use of informal interpreters  No formal validity or reliability checks.  -Coding by few authors may introduce bias.  - Participant diversity low |
| Jones et al., (2022) ¹  (Report) | United Kingdom | 313 service users  Data available for 303 (SU)  Females – 77  (including 7 pregnant women)  Males- 226  Origin: Just under 95% were non-EU people seeking asylum; countries of origin not fully detailed | -To provide evidence on health status and access to healthcare for people seeking asylum residing in initial/contingency accommodation in the UK, including hotels and barracks  -To explore health needs, barriers to healthcare, and the influence of structural and social factors on health | -Mixed methods study combining quantitative and qualitative data collection  - Data sources included DOTW UK standard social and medical questionnaires, free-text case notes, and remote & face-to-face consultations  -Data analysed using descriptive univariate and bivariate statistics; thematic analysis of qualitative data | None | Convenience sampling of people seeking asylum | -Barriers to GP registration  -Poverty  - Isolation  - Accommodation conditions  -Lack of information and continuity of care | Diabetes was mentioned among the reported health conditions people seeking asylum sought help for | Intersectionality was implicitly considered by acknowledging multiple compounding vulnerabilities: immigration status, language, gender (e.g., pregnant women), age (children), housing conditions, and administrative barriers.  - The study involved National Health Advisors with lived experience to inform findings, highlighting intersectional challenges faced by people seeking asylum | - Non-random convenience sample of service users limits generalizability  -Some questionnaires missing complete data (max 313/380 with consent for research use)  -Qualitative data sample was 33% of total for thematic analysis, potentially missing some perspectives |
| Correa-Velez et al., (2008) | Australia | 341 people seeking asylum attending 3 Melbourne clinics, data from 998 consultations (2005-06)  -Males: 187  -Females: 144  -Unknown: 10 | To audit reasons for encounter, diagnostic tests, treatments, and referrals for asylum seekers | Retrospective audit of medical files | No mention the use of a specific theoretical framework. Used a standardized classification system, the Australian version of the International Classification of Primary Care, second edition (ICPC-2 Plus), to categorize reasons for encounters and health issues among people seeking asylum | Convenience sampling of attendees of 3 clinics | -Poverty  - Homelessness  -Social isolation  -Immigration-related issues (such as visa status)  -Problems related to housing  -Difficulties accessing health care  - Limitations on right to work  - Food insecurity  - Financial constraints | -Foot or toe symptoms or complaints accounted for 2.7 per 100 encounters  - Diabetes accounted for 4.5 per 100 encounters | No | -The sample may not be representative of all asylum seekers in Victoria  - The coding process using the ICPC-2 Plus system is complex and may have caused some miscoding of data in a small number of cases  - Some reasons for health care encounters could be classified into more than one category, potentially affecting coding accuracy |
| Phillimore et al., (2025) ¹ | United Kingdom | 313 service users (SUs) living in institutional housing who consulted with DOTW UK and consented to data use for research.  Data available for 303 (SU)  Females – 77  (including 7 pregnant women)  Males- 226  No breakdown of countries or origin but the majority of SUs were classified as non-EU people seeking asylum, accounting for just under 95% of the sample | To analyse the health status and healthcare access of people seeking asylum living in institutional housing using a structural violence framework  To highlight barriers to healthcare and the impact of institutional living conditions on physical and mental health | Mixed methods:  Quantitative data collected through standardized DOTW UK questionnaires (administrative, social, medical) Qualitative data via volunteers' free-text notes documenting consultations and context  Post-hoc secondary data analysis | Structural violence, specifically concepts of slow violence and violent abandonment | Convenience sample of people seeking asylum who engaged with DOTW UK services | - Overcrowding, poor housing conditions, isolation, lack of support (e.g., difficulty registering with GP)  -Language barriers and lack of knowledge about healthcare systems  - Mental health deterioration linked to 'violent abandonment' through inaction and neglect  -Limited access to medication and healthcare services, refusal or incapacity of hotel staff to assist in urgent health needs | Diabetes mentioned as one of the health conditions reported among service users (SUs) requiring medical consultation | No | -Questionnaires not standardized or validated  -Small sample size and indirect data collection via volunteers  - Data not openly available due to confidentiality, limiting reproducibility  -Excludes individuals unable to access or contact DOTW UK services |
| British Red Cross (2021)  (Report) | United Kingdom | Over 100 people seeking asylum (men and women)  Countries of origin not comprehensively listed, but includes individuals from conflict-affected countries such as Syria and Eritrea | To document the lived experiences of people seeking asylum in UK asylum accommodation and advocate for reforms that ensure safety, dignity, and proper healthcare | -Rapid review  -Qualitative methods including interviews, focus groups, and frontline staff insights  British Red Cross case records and testimonies | None | Purposive sampling of people living in various forms of asylum accommodation (e.g. hostels, hotels, military barracks) supported by the British Red Cross. | -Prolonged stays in inappropriate accommodation  -Delays in asylum decisions  -Barriers to healthcare access  -Deterioration in mental health  -Lack of financial autonomy  -Poor safeguarding and neglect of special needs  -Isolated and restricted movement | “We spoke to one man who is paralysed in one leg and requires special adaptations in his room to live independently. After raising concerns about the first hostel he was placed in, he was moved to another accommodation which did not have the adaptations he needs, leaving him unable to use the bathroom and afraid of falling” | The report highlights specific vulnerabilities (e.g., survivors of trafficking, people with disabilities, single mothers), but does not apply a structured or explicit intersectional framework. | -No detailed demographic or clinical breakdown of participants  -Primarily advocacy-focused rather than empirical research |
| Refugee Council (2022)  (Report) | United Kingdom | People seeking asylum accommodated in hotels and other contingency accommodation | To investigate and update on the experiences and challenges faced by people seeking asylum living in hotel accommodation in the UK.  To assess progress since a previous report and provide recommendations. | Mixed methods: analysis of Refugee Council service data and casework; Monitoring, Evaluation & Learning (MEL) study of 39 escalated cases; Freedom of Information (FOI) requests; desk-based research and stakeholder engagement. | None | Convenience sampling based on clients supported by Refugee Council services; 6,241 people supported in 2021, with more than 3,000 in early 2022. MEL study based on 39 escalated cases. | -Accommodation in hotels  -limited access to healthcare, legal and educational services  - mental health challenges  - experiences of safeguarding risks  -food insecurity  - social isolation | - Prolonged stays in hotel accommodation exacerbate health problems, including diabetes management, due to poor access to healthcare services, inadequate nutrition, and lack of appropriate support | No | -Small MEL sample size (39 cases) for detailed case escalation  -data largely from clients engaged with Refugee Council services which may not represent all people seeking asylum |

¹Both papers draw on the same dataset collected by Doctors of the World UK between July 2020 and January 2022 from 313 individuals seeking asylum housed in hotels and barracks. However, they differ in purpose and analytical lens. Jones et al., (2022) present descriptive findings aimed at policy and service improvement, whereas Phillimore et al., (2025) offer a post-hoc academic analysis using a structural violence framework to examine how institutional accommodation actively produces health harms.
