# Supplementary material for: Diabetes and Foot Health Among South Asian People Seeking Asylum in the United Kingdom: A Theory‐Informed Scoping Review
Source: Health Expect. 2026 Jun 18;29(3):e70728. doi: 10.1111/hex.70728 (PMC13280213; doi:10.1111/hex.70728)

Appendix S4

Example of coding and mapping findings to the socioecological model levels

The table below provides an illustrative example of how findings extracted from included studies were coded and mapped to levels of the socioecological model used in this review.

Extracted data describing diabetes experiences and post-migration conditions were summarised and assigned codes. These codes were subsequently organised according to the socioecological model levels to identify the level at which each influence operated. This example demonstrates the analytic process used to generate the themes presented in Figure 4.


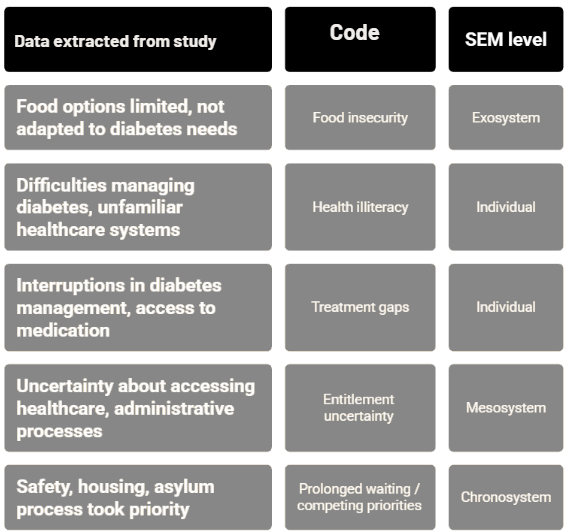

Supplement: Supplementary file 4 — Supporting File 4 [file HEX-29-e70728-s004.docx]
